# Combined Analysis of Transcriptome and Metabolome Reveals the Heat Stress Resistance of Dongxiang Wild Rice at Seedling Stage

**DOI:** 10.3390/plants14081192

**Published:** 2025-04-11

**Authors:** Peng Zhang, Haipeng Yu, Zengying Huang, Pengfei Yang, Huijuan Li, Guanrong Huang, Lu Tang, Zhengzheng Zhong, Guocheng Hu, Guoping Yu, Hanhua Tong

**Affiliations:** 1 State Key Laboratory of Rice Biology and Breeding, China National Rice Research Institute, Hangzhou 310006, China; yuhaipengcnrri@163.com (H.Y.); ypf19990703@163.com (P.Y.); lihuijuan0812@163.com (H.L.); 13417992759@163.com (G.H.); zhongzhengzheng@caas.cn (Z.Z.); huguocheng@caas.cn (G.H.); 2 National Nanfan Research Institute, Chinese Academy of Agricultural Sciences, Sanya 572024, China; yuguoping@caas.cn; 3 State Key Laboratory for Conservation and Utilization of Subtropical Agro-Bioresources, College of Agriculture, South China Agricultural University, Guangzhou 510642, China; tenzyiii@163.com (Z.H.); 18570360851@163.com (L.T.)

**Keywords:** Dongxiang wild rice, heat stress, transcriptomics, metabolomics, combined analysis

## Abstract

Rice is sensitive to high temperatures at the seedling stage. In the present study, a combined analysis of transcriptome and metabolome was performed on a heat-resistant accession, DY80, from Dongxiang wild rice and a heat-sensitive variety, R974, under heat stress at the seedling stage. The results of the transcriptome and metabolome analyses were verified through qRT-PCR and ultra-performance liquid chromatography–tandem mass spectrometry (UPLC-MS/MS) analysis. We found that there were 1817 and 561 differentially expressed genes (DEGs) unique in DY80 and R974 under heat stress, respectively. The elite genes for the heat stress involved in Dongxiang wild rice may include upregulated genes in the pathway of unfolded protein binding; downregulated genes in the pathways of chlorophyll biosynthetic process, and cysteine and methionine metabolism; and photosystem I, photosystem II, and unchanged genes in the pathways of the anchored component of the plasma membrane, cell wall biogenesis, and photosynthesis-antenna proteins. Moreover, a total of 301 and 28 metabolites were identified as unique in DY80 and R974 after heat treatment, respectively. Further analyses showed that malic acid, stearic acid, and L-threonine might be causal metabolites, contributing to strong heat resistance in Dongxiang wild rice. These findings provide new insights into the mechanisms of heat resistance in rice.

## 1. Introduction

Rice is one of the most important staple crops worldwide, for more than half of the global food demand. Particularly in Asia, rice provides 80% of the caloric necessities of the population. However, greenhouse gases are already causing a steady rise in global temperature. The sixth assessment report of the Intergovernmental Panel on Climate Change (IPCC) indicates global surface temperatures were about 1.1 °C higher in 2011–2020 than in 1850–1900. The frequency and intensity of extreme heat events have been increasing in the vast majority of the world since the 1950s “https://www.ipcc.ch/data/ (accessed on 9 August 2021)”.

Rice is one of the crops most vulnerable to the effects of global warming. It is most sensitive to high temperatures, particularly in the heading and filling stages of rice, and can lead to severe yield reductions [1]. Rice yields would decrease by an average of 3.2% for every 1.0 °C increase in the global average temperature [2]. Heat stress has a damaging effect on the metabolic processes at all stages of rice growth [3]. Rice could respond to heat stress like other plants by activating downstream responses at different levels under heat stress, including disruptions in cell membrane fluidity [4,5], protein homeostasis [6], and reactive oxygen species homeostasis [7]. Furthermore, a large number of heat-related genes have been cloned in rice, e.g., *OsHSP17.7* [8], *OsMYB55* [9], *OsHsfA2a* [10], *HSA32* [11], *OsHsfA2d* [12], *SNAC3* [13], *OsTT1* [1], *OsHTAS* [14], *SLG1* [15], *TT2* [16], *TT3.1* [17], and *OsGRP3/OsGRP162* [18]. These studies suggest that the response to high temperatures in rice is regulated by multiple genes, and the mechanism of responding to high temperatures is quite complex.

The combined analysis of multi-omics is an efficient approach to achieving a full spectrum of genes and phenotypes. Transcriptomics can reveal differentially expressed genes (DEGs) under different conditions, and other methods like metabolomics, biochemical, cytometric, as well as proteomics are the bridge between genes and phenotypes. Combined analyses could provide more comprehensive insights into the mechanisms of stress resistance in plants. Furthermore, γ-aminobutyric acid (GABA) was induced by high temperatures in rice by combining transcriptomics and metabolomics analyses [19]. The mechanism of zinc homeostasis and antioxidant machinery was revealed in tobacco responding to different zinc supplies through a combined transcriptome and proteome analysis [20]. Volatile substances and plant kinetic changes in sweet corn seedlings were analyzed at extreme temperatures by a combined transcriptomics and metabolomics analysis [21]. The regulatory network was revealed by using integrated flow cytometric and proteomics analyses in sugarcane protoplast responses to fusion [22]. Phosphate-starving tolerance was uncovered in two ramie varieties using comparative biochemical and transcriptomics analyses [23].

Dongxiang wild rice is a common wild rice (*Oryza rufipogon* Giff.) that originated from Dongxiang County (28°14′ N, 116°36′ E) of Jiangxi Province in China. Dongxiang wild rice is the northernmost *O. rufipogon* that has been found to date [24,25]. Previous studies have shown that Dongxiang wild rice contains a great many elite genes/alleles, which allow it to survive under extreme abiotic stresses, e.g., drought [26,27,28,29], cold [30,31], and salt [30]. However, the QTL/genes from Dongxiang wild rice that are potentially responsible for improving heat stress have not been reported.

The mechanism of heat tolerance in rice needs to be further studied. In the present study, we aim to mine novel elite genes and identify the key metabolic pathways relating to heat stress in Dongxiang wild rice through combined transcriptomics and metabolomics analyses. The results of the present study could shed light on improving heat resistance in rice and other crops.

## 2. Results

### 2.1. Performance of DY80 and R974 Under Heat Stress

**Figure 1:**
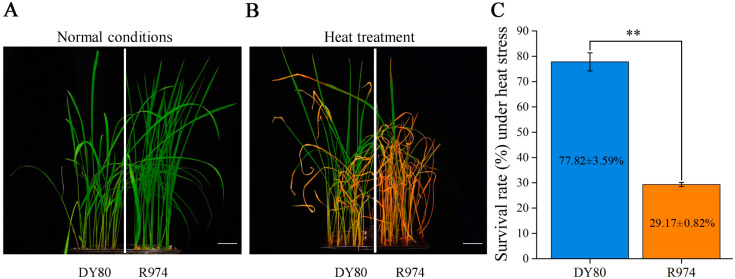
Performance of DY80 and R974 under normal and heat stress conditions at the seedling stage: (**A**) Growth statement in normal conditions for 15.5 days; bar = 3.0 cm. (**B**) Growth statement for recovering seven days after 36 h heat treatment; bar = 3.0 cm. (**C**) Survival rate of two varieties for recovering seven days after 36 h heat treatment. ** represents a *p* < 0.01 level using the Student’s *t*-test. *n* = 48, which represents the number of plants used to calculate the survival rate under heat stress in each replication.

After 36 h under heat stress (45 °C with light and 42 °C without light) for DY80 and R974, the two varieties showed a significant difference in leaf wilting and curling. As shown in Figure 1, about one-third of the second and third leaf areas wilted for most of the seedlings of DY80; however, only a portion of the leaf sheaths of R974 seedlings remained green, while all other seedlings died. The survival rate of DY80 under heat stress was 77.82%, while that of R974 was much lower. The results indicated that DY80 was more heat-tolerant than R974.

### 2.2. Transcriptome Sequencing

To investigate the differences in gene expression between DY80 and R974 under heat stress, the leaves of the two varieties after 36 h under heat stress and normal conditions were collected for transcriptome sequencing. A total of 73.36 Gb of clean data were obtained, with a minimum of 93.11% of clean data achieving a quality score of Q30 (Table S1). The mapping ratio of each sample against the reference genome ranged from 93.24% to 94.66% (Table S2). These results indicated that the quality of transcriptome sequencing data is suitable for the following analyses.

**Figure 2:**
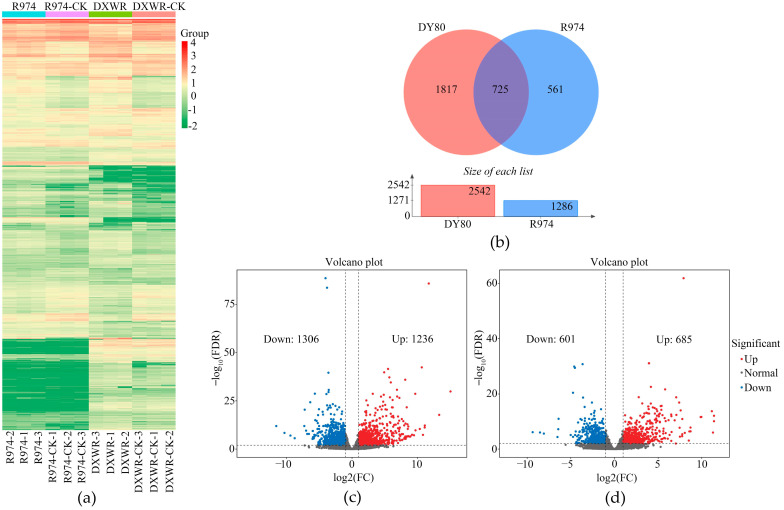
Transcriptomic analyses of DY80 and R974 under heat stress at the seedling stage: (**a**) Expression patterns of differentially expressed genes (DEGs) of DY80 and R974 with V.S. without heat stress. DXWR represents DY80, CK represents without heat treatment. (**b**) Venn diagram showing the number of DEGs of DY80 and R974 with V.S. without heat stress, and (**c**,**d**) are the volcano plots of DEGs of DY80 and R974 with V.S. without heat stress, respectively. FDR represents a false discovery rate.

PCC and PCA were performed to verify three biological replications, and the results of the above two analyses indicated a high correlation among the three biological replications (Figure S1). Moreover, the heatmap also showed an identical truth with PCC and PCA (Figure 2a).

### 2.3. Gene Expression Patterns in DY80 and R974 After Heat Treatment

DY80 and R974 had significantly different expression patterns before and after applying heat stress (Figure 2a). There were 1236 upregulated and 1306 downregulated DEGs in DY80 after heat treatment (Table S3), while 685 upregulated and 601 downregulated DEGs were in R974 (Table S4, Figure 2b–d). There were 1817 and 561 DEGs that were unique in DY80 and R974, respectively (Figure 2b). Furthermore, the number of DEGs of DY80 being annotated based on the databases of Nr, Pfam, COG, KOG, Swiss-Prot, KEGG, and GO were significantly larger than those of R974 (Table S5 and Figure S2). The results indicated that heat stress played an important role in gene expression in rice and that the transcriptomics data were significantly different between DY80 and R974.

Furthermore, GSEA, based on the KEGG and GO analyses, showed that three identical pathways in DY80 and R974, i.e., nucleosome, apoplast, and protein processing in the endoplasmic reticulum, were found in DY80 and R974 after heat treatment (Figure S3). However, the chlorophyll biosynthetic process, cysteine and methionine metabolism, photosystem I, photosystem II, and unfolded protein binding were unique in DY80 after heat treatment (Figure S4a–e), while three pathways (i.e., the anchored component of the plasma membrane, cell wall biogenesis, and photosynthesis-antenna proteins) were found significantly differential in R974 after heat treatment (Figure S4f–h). Furthermore, most of genes in the pathways of the nucleosome, apoplast, chlorophyll biosynthetic process, cysteine, methionine metabolism, photosystem I, photosystem II, anchored component of the plasma membrane, cell wall biogenesis, and photosynthesis-antenna proteins were downregulated significantly after heat treatment, while those in the pathways of protein processing in the endoplasmic reticulum and unfolded protein binding were upregulated significantly (Figures S3 and S4).

### 2.4. Verification of Transcriptomic Sequencing by qRT-PCR

**Figure 3:**
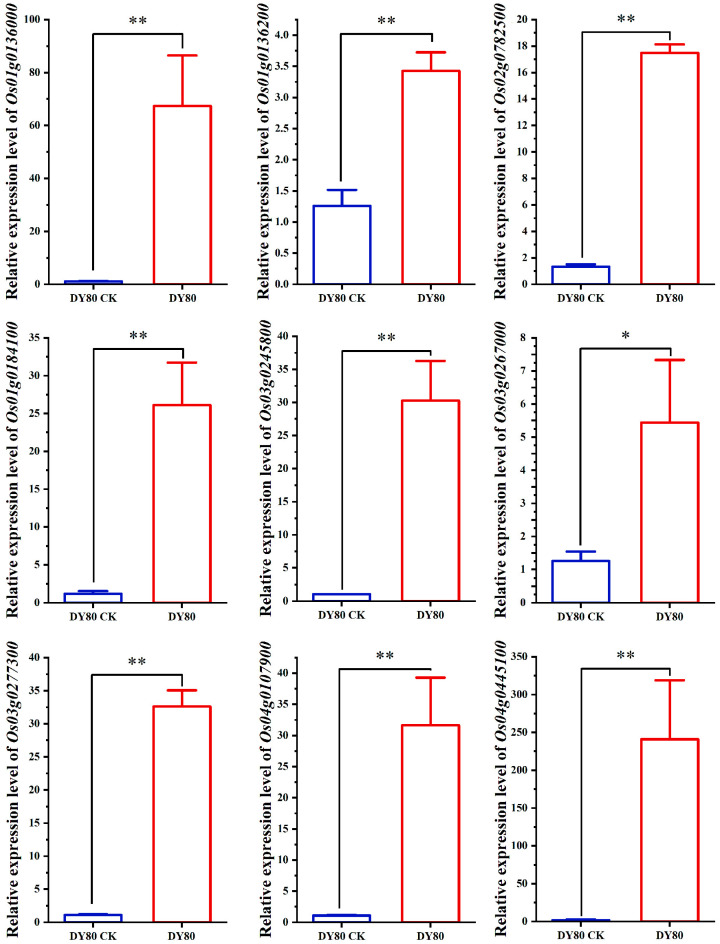
Expression of nine candidate DEGs in DY80 with V.S. without heat stress. * and ** represent significant differences at the *p* < 0.05 and *p* < 0.01 levels (Student’s *t*-test.), respectively. CK represents without heat treatment.

Nine genes that encode heat-shock proteins with significantly upregulating expressions after the heat treatment only in DY80 were selected randomly. The results of the qRT-PCR for the above nine genes showed that their expressions were upregulated, ranging from about 3 to 200 folds by heat treatment (Figure 3).

### 2.5. Analyses of Differential Metabolites After Heat Treatment in DY80 and R974

**Figure 4:**
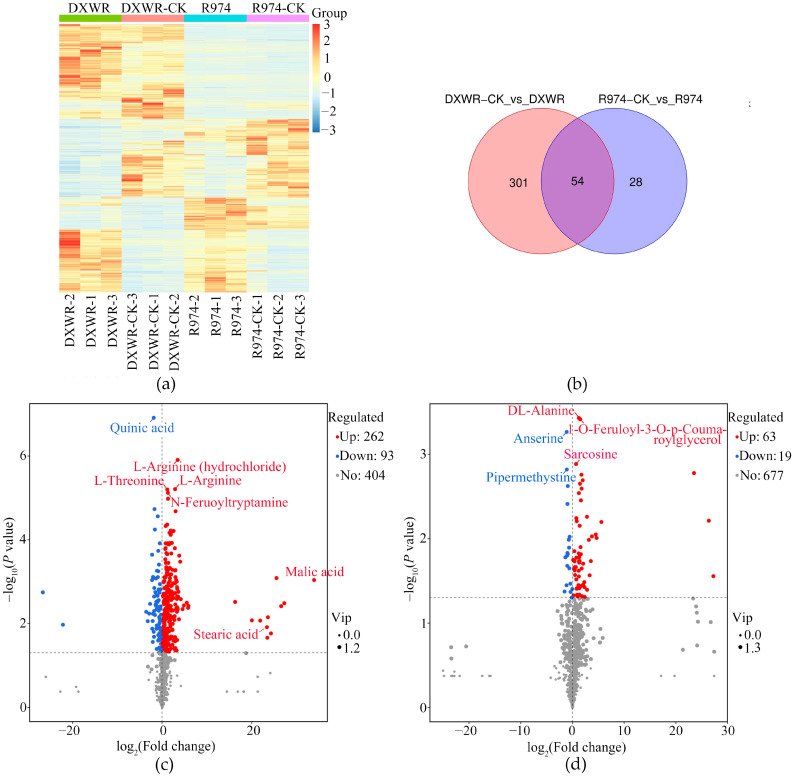
Metabolomic analyses of DY80 and R974 under heat stress at the seedling stage: (**a**) Cluster heat map of differential metabolites in DY80 and R974 with V.S. without heat stress. DXWR represents DY80, and CK represents without heat treatment. (**b**) Venn diagram showing the numbers of differential metabolites in DY80 and R974 with V.S. without heat stress. DXWR represents DY80, and CK represents without heat treatment, and (**c**,**d**) are volcano plots of differential metabolites in DY80 and R974 with V.S. without heat stress, respectively. The purple rectangles in (**c**) represent the metabolites selected for being verified.

Moreover, we found that the *r*-values ranged from 0.766 to 0.970, which indicates a high correlation among the three biological replications (Figure S5a). Moreover, PCA was performed on two samples to determine the separation trend of the metabolites between groups and whether there were differences in the metabolites within groups. The analysis showed that there was a clear separation trend between DY80 and R974 (Figure S5b). At the same time, we also carried out a cluster hierarchical analysis, which showed obvious differential metabolomic patterns between the two varieties (Figure 4a).

Furthermore, we found that a total of 355 and 82 metabolites were significantly detected after the heat treatment based on an UPLC-MS/MS detection platform and self-built database for DY80 and R974, respectively (Figure 4b, Table 1, Tables S6 and S7). Moreover, there were 262 and 63 upregulated significant metabolites in DY80 and R974, while 93 and 19 significant downregulated metabolites were identified in DY80 and R974 after heat treatment, respectively (Figure 4b and Table 1). A further analysis showed that differential metabolites were detected between DY80 and R974 after heat treatment, e.g., malic, stearic acid, and L-threonine were detected uniquely in DY80 (Figure 4c,d, Tables S6 and S7).

### 2.6. Combined Analyses of Differential Metabolites and DEGs

**Figure 5:**
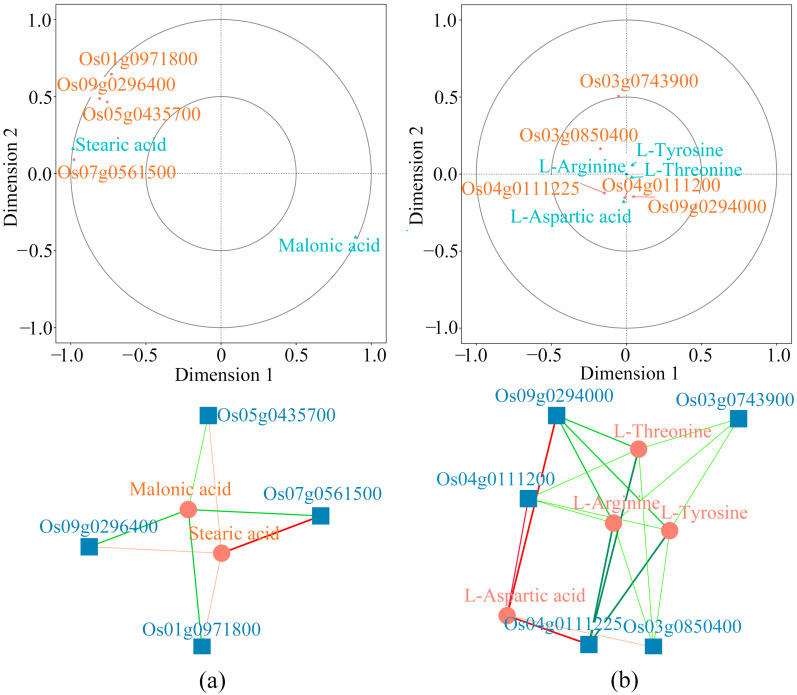
Combined analyses of transcriptomic and metabolomic data of the KEGG pathways in DY80 and R974: (**a**) Pathway of fatty acid biosynthesis, and (**b**) pathway of glycine, serine, and threonine metabolism. In the top panels, an orthogonal partial least squares discriminant analysis (OPLS-DA) was performed for DEGs and differential metabolites. Dimensions 1 and 2 represent the predictive and orthogonal components, respectively. The bigger and smaller circles represent 95.0% and 99.0% confidence, respectively. In the bottom panels, the blue squares represent DEGs, and the orange circles represent metabolites. The orange line represents a positive correlation between the DEGs and metabolites, while the green line represents a negative correlation between the DEGs and metabolites.

After identifying the differential metabolites between DY80 and R974 under heat treatment, combined analyses between the differential metabolites and DEGs were performed using BMKCloud (www.biocloud.net (accessed on 9 August 2021)) based on the annotations of DEGs and the KEGG database. Interestingly, there were four genes, i.e., *Os09g0296400*, *Os05g0435700, Os07g0561500*, and *Os01g0971800*, that associated positively with stearic acid and negatively with malonic acid (Figure 5a). Moreover, there were five genes, i.e., *Os03g0743900*, *Os04g0111225*, *Os03g0850400*, *Os04g0111200*, and *Os09g0294000*, that associated negatively with L-threonine, L-arginine, and L-tyrosine, while four of the above five genes were associated positively with L-aspartic acid (Figure 5b). Moreover, it was found that *Os09g0296400*, *Os05g0435700*, *Os07g0561500*, *Os03g0743900*, *Os04g0111225* and *Os04g0111200* were unique DEGs in DY80 (Tables S3 and S4).

**Figure 6:**
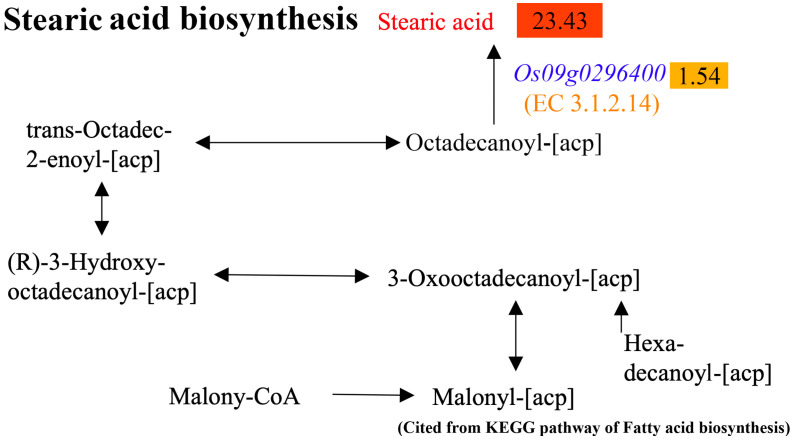
Alteration of stearic acid biosynthesis in the pathway of fatty acid biosynthesis in DY80 with V.S. without heat stress. The positive values in the rectangles represent the significantly upregulated metabolites and genes identified by metabolome and transcriptome analyses, respectively.

A further analysis showed that *Os09g0296400* encoded an enzyme numbered EC 3.1.2.14, which directly determines octadecanoyl-[acp] to be stearic acid in the pathway of stearic acid biosynthesis (Figure 6). Moreover, it was found that *Os05g0435700* encodes a (3R)-hydroxymyristoyl-[acyl carrier protein] dehydratase, and *Os07g0561500* encodes a protein containing short-chain dehydrogenase/reductase (SDR) domains, which are involved in the pathway of fatty acid biosynthesis (The Rice Annotation Project Database and KEGG database). We speculated that heat may induce the upregulation of stearic acid by upregulating *Os05g0435700* and *Os07g0561500* in DY80.

**Figure 7:**
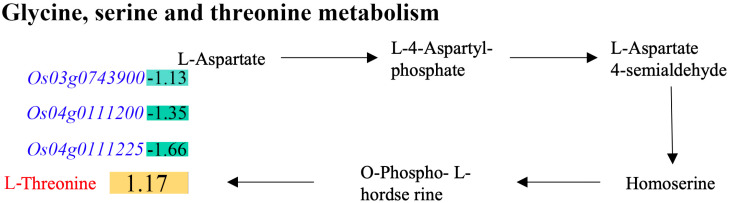
Alteration of L-threonine biosynthesis in the pathways of glycine, serine, and threonine metabolism in DY80 with V.S. without heat stress. The positive values in the rectangles represent the significantly upregulated metabolites and genes identified by metabolome and transcriptome analyses, respectively, while the negative values represent the significantly downregulated ones.

Moreover, L-threonine was upregulated when three genes were downregulated in DY80 after the heat treatment, i.e., *Os03g0743900*, *Os04g0111200*, and *Os04g0111225* (Figure 7, Tables S3 and S6). More interestingly, it has been discovered that the aforementioned three genes belong to the same gene family, which encodes an ATP sulfurylase (The Rice Annotation Project Database).

### 2.7. Verification of the Association Between Transcriptome and Metabolome Analyses

**Figure 8:**
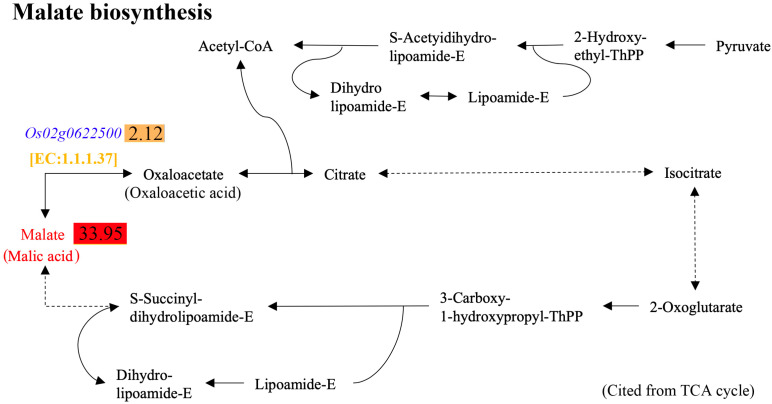
Alteration of malic acid biosynthesis in the tricarboxylic acid (TCA) cycle pathway in DY80 with V.S. without heat stress. The positive values in the rectangles represent the significantly upregulated metabolites and genes identified by metabolome and transcriptome analyses, respectively.

**Figure 9:**
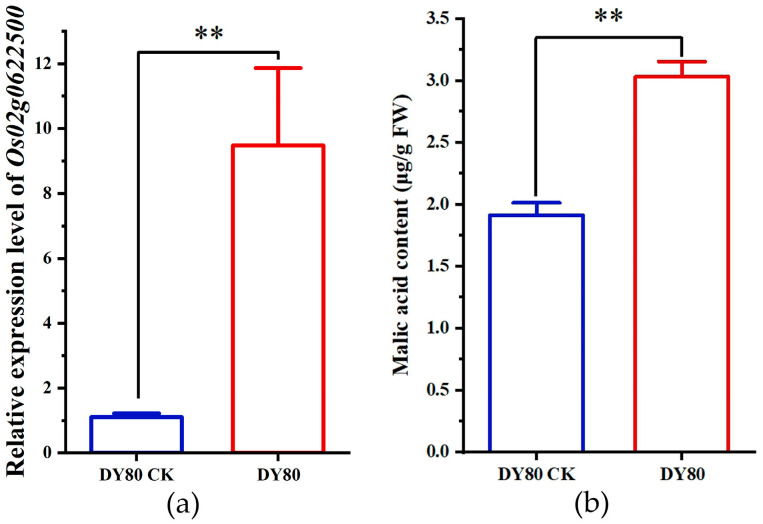
Verification of malic acid: (**a**) Alteration of *Os02g0622500* expression in DY80, and (**b**) alteration of malic acid content in DY80. CK represents without heat treatment, FW represents fresh weight, and ** represents a *p* < 0.01 level using the Student’s *t*-test.

Malic acid was upregulated by 33.95 folds in DY80 after heat treatment, while there was no significant change in R974 (Figure 8, Tables S6 and S7). A further analysis showed that one upregulated gene, called *Os02g0622500*, encodes an enzyme numbered EC 1.1.137, which plays an important role in turning oxaloacetate into malic acid (Figure 8 and KEGG database). Due to no evidence by the combined analyses for transcriptome and metabolome using BMKCloud (www.biocloud.net (accessed on 9 August 2021)), differences between the *Os02g0622500* expression level and malic acid content in DY80 before and after heat treatment were detected by qRT-PCR and UPLC-MS/MS analyses, respectively. The results showed that both the *Os02g0622500* expression level and malic acid were upregulated after heat treatment in DY80 (Figure 9).

## 3. Discussion

It is easy to understand that Dongxiang wild rice might contain elite genes or alleles for cold stress due to its position as the northernmost wild rice. Hence, there is no research examining whether Dongxiang wild rice is a good donor of elite genes or alleles for heat stress. In the present study, DY80, an accession of Dongxiang wild rice, showed strong resistance to extreme heat stress. It will be very useful to utilize the elite genes or alleles from Dongxiang wild rice for abiotic resistance genetic improvement in rice because Dongxiang wild rice has been proven to contain elite resistant genes or alleles to drought, cold, salt, and low phosphorus.

There were three common KEGG pathways showing a significant differential between DY80 and R974 after heat treatment, i.e., nucleosome, apoplast, and protein processing in the endoplasmic reticulum. Nucleosomes have been proven to affect the transcription of genes that are related to heat stress in previous studies. For instance, nucleosomes during heat stress in yeast are displaced from heat-stress-dependent promoters during the activation of transcription, and the chromatin-remodeling complexes SWI/SNF, ISW1, and RSC seem to have partially overlapping functions in this process [32,33,34]. Moreover, it has been discovered that heat-induced electrical signals affect both cytoplasmic and apoplastic pH in maize leaves [35]. Our results in Figure S3 showed that most genes involved in the pathway of protein processing in the endoplasmic reticulum were significantly upregulated, which was identical to the results in a previous study [36].

Different gene expression patterns of DY80 and R974 with heat treatment were also uncovered by the transcriptomic analysis. In the present study, five pathways, i.e., the chlorophyll biosynthetic process, cysteine and methionine metabolism, photosystem I, photosystem II, and unfolded protein binding, were uniquely identified in DY80 after heat treatment. The above results were supported by previous studies. For the chlorophyll biosynthetic process, chill and heat stress could affect cucumber and wheat seedlings due to impairment of 5-aminolevulinic acid biosynthesis in the pathway of the chlorophyll biosynthetic process, respectively [37]. For cysteine and methionine metabolism, the over-synthesis of methionine was found in mature leaf cells of *Rhazya stricta* due to the activation of methionine synthase, tyrosine amino-transferase, and aromatic-amino-acid transaminase under heat stress [38]. For photosystem I, heat stress could lead to the induction of cyclic electron flow around photosystem I in grape leaves [39]. For photosystem II, a previous study found that it was sensitive to heat stress in soybean [40]. For unfolded protein binding, the expressions of coding the unfolded protein response sensors, *OsIRE1*, *OsbZIP39*/*OsbZIP60*, and the unfolded protein response marker *OsBiP1*, were upregulated under heat stress in rice [12]. Heat-shock proteins have been widely recognized as being associated with plant heat tolerance [11,12,32,41]. In the present study, nine upregulated DEGs coding heat-shock proteins between DY80 and R974 were identified through transcriptomic analysis and were verified by qRT-PCR analysis. These nine heat-shock protein-related genes may be one of the reasons for DY80’s strong heat tolerance and are worthy of focused research in the future. Moreover, an exclusive focus on upregulated DEGs raises concerns, as downregulated DEGs may also provide valuable insights into heat stress tolerance, particularly in R974.

Three pathways, i.e., the anchored component of the plasma membrane, cell wall biogenesis, and photosynthesis-antenna proteins, were found to be differentially significant in R974 after heat treatment. For the anchored component of the plasma membrane, a previous study proved that the plasma membrane could be the first responder to heat stress [41]. For cell wall biogenesis, heat stress could lead to alternations in cell wall polymers and anatomy in coffee leaves [42]. For photosynthesis-antenna proteins, heat stress could cause inhibition of the de novo synthesis of antenna proteins and photobleaching in cultured *Symbiodinium* [43].

Summarily, the elite genes for heat stress in Dongxiang wild rice might be those upregulated in the unfolded protein binding pathway, downregulated in the pathways of chlorophyll biosynthetic process, cysteine and methionine metabolism, photosystem I, photosystem II, and unchanged in the pathways of anchored component of the plasma membrane, cell wall biogenesis, and photosynthesis-antenna proteins.

In the present study, 301 and 28 metabolites were uniquely detected in DY80 and R974 after heat treatment, respectively. Three unique metabolites in DY80, i.e., malic acid, stearic acid, and L-threonine, which were proven to associate with heat stress, were selected for combined transcriptome and metabolome analyses for verification.

In previous studies, malic acid was not proven to be associated directly with heat stress in plants. However, reactive oxygen species (ROS) are elevated in chloroplasts under stressful environments, which leads to changes in cell function to adjust to the effects of excess reducing equivalents [44]. Malate circulation links chloroplast metabolism to mitochondrial ROS [45], and ROS equivalents caused by stress could be reduced through malate circulation [46]. Our study found that malic acid was significantly upregulated in DY80 after heat treatment, while there was no change in R974, and the expression of *Os02g0622500* encoding malate dehydrogenase was upregulated significantly. Thus, malic acid might be one of the causal metabolites for strong resistance to heat stress in Dongxiang wild rice.

Stearic acid might play an important role in resistance to heat stress within Dongxiang wild rice. Reducing the degree of fatty acid unsaturation is one of the essential adaptation strategies that is followed by plants to cope with heat stress [5], and stearic acid belongs to saturated fatty acid. A stearic acid desaturase gene, *PtSAD*, is involved in the thermal stress response [47]. Our study showed that the upregulation of *Os09g0296400* might be responsible for stearic acid’s upregulation in DY80 after heat treatment because *Os09g0296400* encodes an enzyme, numbered EC 3.1.2.14, which determines octadecanoyl-[acp] to be stearic acid in the pathway of stearic acid biosynthesis.

Moreover, L-threonine might be another causal metabolite for strong resistance to heat stress in Dongxiang wild rice. L-threonine induces heat-shock protein expression and decreases apoptosis in heat-stressed intestinal epithelial cells [48]. In the present study, L-threonine was upregulated in DY80 after heat treatment when *Os03g0743900*, *Os04g0111200,* and *Os04g0111225* were downregulated in our study. The first reason for the inconsistencies between genes and metabolites may be that post-transcriptional or post-translational regulation may be involved in the L-threonine pathway because post-transcriptional regulation has been shown to play a role in the fine-tuning of gene expression or protein function [49]. Moreover, the aforementioned three genes belong to the same gene family, which encodes an ATP sulfurylase (The Rice Annotation Project Database). ATP sulfurylase is involved in sulfur assimilation, providing sulfate for the synthesis of cysteine [50]. There may be a competitive relationship between the synthesis of L-threonine and cysteine. The activity of ATP sulfurylase decreases when *Os03g0743900*, *Os04g0111200*, and *Os04g0111225* are downregulated, which leads to the inhibition of cysteine synthesis, thereby indirectly promoting threonine synthesis.

In summary, this study provides valuable insights into the molecular and metabolic mechanisms underlying heat resistance in rice at the seedling stage, particularly through the comparative analyses of heat-resistant Dongxiang wild rice accession DY80 and the heat-sensitive variety R974. However, several limitations should be acknowledged to guide future research. Firstly, while the transcriptome and metabolome analyses revealed significant differences in gene expression and metabolite profiles between DY80 and R974, this study focused on a single time point under heat stress. Future studies should incorporate multiple time points to capture the dynamic changes in gene expression and metabolite levels, which could provide a more comprehensive understanding of the temporal regulation of heat stress responses. Secondly, although this study identified the key genes and metabolites associated with heat resistance, the functional validation of these candidates remains limited. Further research should employ genetic engineering techniques, such as CRISPR/Cas9-mediated gene knockout or overexpression, to confirm the roles of the identified genes (e.g., those involved in unfolded protein binding, chlorophyll biosynthesis, and cysteine and methionine metabolism) in conferring heat tolerance. Similarly, the causal relationship between the identified metabolites (e.g., malic acid, stearic acid, and L-threonine) and heat resistance should be experimentally validated through targeted metabolic engineering or exogenous application studies. Another limitation is the focus on only two rice accessions, which may not fully represent the genetic diversity of heat stress responses in rice. Expanding the study to include a broader range of heat-resistant and heat-sensitive varieties, as well as different wild rice species, could help to identify the additional genetic and metabolic markers associated with heat tolerance. Additionally, this study was conducted under controlled environmental conditions, which may not fully replicate field conditions. Field trials under natural heat stress conditions are necessary to validate findings and assess their practical applicability in breeding programs.

## 4. Materials and Methods

### 4.1. Plant Material

The survival rate under heat stress in the seedling stage was used to evaluate the heat tolerance of one rice variety; please see more details in the studies of Prof. Hongxuan Lin [1,16]. DY80, an accession of Dongxiang wild rice, and R974, an *indica* restorer line, were chosen for combined transcriptomic and metabolomic analyses under heat stress in the present study.

### 4.2. Sample Preparation

The heat treatment was conducted in an incubator (full intelligent artificial climate plant box, HP1500 GS-B). The uniform seeds were sterilized with 1% H_2_O_2_ solution for 30 min and then were washed three times with deionized water. Sterilized seeds were spread in Petri dishes with qualitative filter papers and were soaked in deionized water to be germinated in an incubator at 30 °C, and the water was changed every 12 h. After 36 h, the germinated seeds were moved to a 37 °C dark incubator to promote root elongation. Uniform, well-germinating seeds were selected and transferred into 96-well plates in a 1 L plastic container with conditions of 14 h of light (30 °C, 70.5% RH, 20,000 Lx)/10 h of darkness (28 °C, 70.5% RH, 0 Lx), and were then were cultured with Yoshida solution (pH = 5.65) [51]. We changed the culture solution every 3 days until the seedlings reached the two-leaf stage. Before the heat treatment, DY80 and R974 were divided into two groups as follows: a control group and a heat-treated group. After seven days, the heat-treated group was transferred into an incubator under 14 h of light (45 °C, 80% RH)/10 h of darkness (42 °C, 80% RH) for 36 h, and leaves with or without heat exposure were collected after the heat treatment and were freeze-dried immediately with liquid nitrogen. Three replications were performed. The remaining seedlings were cultured with Yoshida solution in normal conditions for seven days, and the survival rates of DY80 and R974 after the heat treatment were measured.

### 4.3. Transcriptomic Sequencing

Total RNA was extracted using a miRNeasy Kit (QIAGEN, Germantown, MD, USA). The RNA concentration and purity were measured by a NanoDrop 2000 (Thermo Fisher Scientific, Wilmington, DE, USA), and RNA integrity was accurately tested using the RNA Nano 6000 Assay Kit from the Agilent Bioanalyzer 2100 system (Agilent Technologies, Santa Clara, CA, USA). Qualified RNAs were processed for library construction. The procedures are described as follows: (1) mRNA was isolated by Oligo(dT)-attached magnetic beads. (2) mRNA was then randomly fragmented in a fragmentation buffer. (3) First-strand cDNA was synthesized with fragmented mRNA as a template and random hexamers as primers, followed by second-strand synthesis with the addition of PCR buffer, dNTPs, RNase H, and DNA polymerase I. Purification of cDNA was processed with AMPure XP beads. (4) Double-strand cDNA was subjected to end repair. Adenosine was added to the end and ligated to the adapters. AMPure XP beads were then applied to select fragments within the size range of 300–400 bp. (5) The cDNA library was obtained by certain rounds of PCR on cDNA fragments generated from step 4. qRT-PCR was processed to obtain a more accurate library concentration. A library with a concentration larger than 2 nM is acceptable. RNA sequencing was performed by the Illumina NovaSeq 6000 (San Diego, CA, USA) to generate 150 bp paired-end reads. Sequencing and alignment were performed by Biomarker Technologies Co., Ltd. (Beijing, China). DESeq2_EBSeq in the R package was used for quantifying the transcripts and gene expression levels [52]. A log_2_ (fold change, FC) ≥ 2 and a *p*-value < 0.01 were used as the criteria for detecting DEGs between DY80 and R974 after heat stress.

### 4.4. Metabolomic Analysis

Metabolites were extracted from the freeze-dried leaves according to the method described in a previous study [53] before ultra-performance liquid chromatography–tandem mass spectrometry (UPLC-MS/MS) analysis was conducted. The samples were analyzed using the UPLC-ESI-MS/MS system (UPLC, Waters Acquity I-Class PLUS; MS, Applied Biosystems 6500+ Q TRAP), performed by Biomarker Technologies Co., Ltd. (Beijing, China). The analytical conditions and sample measurements gradient program were followed as described in a previous study [53]. The effluent was alternatively connected to an ESI-triple quadrupole-linear ion trap (QTRAP)-MS. The ESI source operation parameters were referred to as those described in a previous study [54]. The BMKGDB (BMK G database, http://www.biomarker.com.cn/biocloud/fenxi (accessed on 9 August 2021)) and MRM (multiple reaction monitoring) were used for qualitative and quantitative analyses of the metabolites, respectively [55].

A log_2_FC ≥ 1, a *p*-value < 0.01, and a value of variable importance in projection (VIP) ≥1 from the OPLS-DA model were used to detect the differentially significant metabolites between DY80 and R974 after heat stress. Significant differential metabolites were identified and annotated using the Kyoto Encyclopedia of Genes and Genomes (KEGG) compound database (http://www.kegg.jp/kegg/compound/ (accessed on 9 August 2021)), and the annotated metabolites were mapped into the KEGG pathway (http://www.kegg.jp/kegg/pathway.html (accessed on 9 August 2021)).

### 4.5. Statistical Analyses

Gene function was annotated based on the following databases: Nr (NCBI non-redundant protein sequences, http://ftp.ncbi.nih.gov/blast/db/ (accessed on 9 August 2021)); Nt (NCBI non-redundant nucleotide sequences); Pfam (Protein family, http://pfam.xfam.org/ (accessed on 9 August 2021)); COG (Clusters of Orthologous Groups of proteins, http://www.ncbi.nlm.nih.gov/COG/ (accessed on 9 August 2021)); Swiss-Prot (A manually annotated and reviewed protein sequence database, http://www.uniprot.org/ (accessed on 9 August 2021)); KO (KEGG Ortholog database, http://www.genome.jp/kegg/ (accessed on 9 August 2021)); and GO (Gene Ontology, http://www.geneontology.org/ (accessed on 9 August 2021)). WEGO 2.0 (http://wego.genomics.org.cn/ (accessed on 9 August 2021)) was used to visualize the ontology of genes with different expression patterns between DY80 and R974 after heat stress.

GO enrichment analysis of the differential expressed genes (DEGs) and gene set enrichment analysis (GSEA) was implemented by the clusterProfiler software in *R* packages. The KOBAS database and clusterProfiler software were used to test the statistical enrichment of differential expression genes in KEGG pathways. Principal component analysis (PCA) and supervised multivariate OPLS-DA were performed on transcriptomics and metabolomics data using statistical functions in *R* software. VIP values were extracted from the OPLS-DA results and were generated using the *R* package. Heat maps were generated by the PheatMap software of the *R* package, and Pearson correlation coefficients (*r*-value) between three biological replications were calculated by the cor function of the *R* package and displayed by PheatMap. Combined transcriptomic and metabolomic analyses were performed using BMKClouds (www.biocloud.net (accessed on 9 August 2021)).

### 4.6. Real-Time Quantitative PCR

Total RNA was extracted from the leaves after 36 h, with or without heat exposure with the miRNeasy Kit (QIAGEN, Germantown, MD, USA). RNA was converted to cDNA by using the ReverTra Ace qRT-PCR Master Mix kit with gDNA remover (TOYOBO, Shanghai, China). The expression was determined with the THUNDERBIRD TM SYBR^®^ qPCR Mix without ROX (TOYOBO, Shanghai, China) by Roche Light Cycler^®^ 480II (Roche, Basel, Switzerland). Nine genes that were significantly upregulated for DY80 after being heat-treated in transcriptomics were selected for qRT-PCR, while there was no significant change for R974. The primer sequences for qRT-PCR are shown in Table S8. *Ubiquitin* was used as an internal control gene. Relative expression levels were calculated with the 2^−∆∆Ct^ method. Three independent replicates were made for each treatment. The volume of the qRT-PCR reaction system was 10 µL as follows: SYBR Premix Ex Taq II, 2×, 5 µL; PCR-primer (Forward + Reverse, 10 µM), 2 µL; cDNA, 2 µL; and ddH_2_O, 1 µL. The profile of the PCR program was 95 °C for 3 min; 45 cycles of 95 °C for 10 s, 58 °C for 15 s, and 72 °C for 25 s; 95 °C for 10 s; 65 °C for 1 min; and 40 °C for 1 min.

### 4.7. Verifying the Result of Combined Transcriptomic and Metabolomic Analyses

The expression level of candidate gene *Os02g0622500* from the leaves after 36 h, with or without heat exposure, in DY80, was verified by qRT-PCR, and the primer sequences for qRT-PCR are shown in Table S8. The qRT-PCR reaction system and the profile of the PCR program are the same as above. Moreover, the malic acid content in DY80, with or without heat treatment, was quantitatively detected by using UPLC-MS/MS analysis, and a standard sample of malic acid was prepared in Shanghai Yuanye Bio-Technology Co., Ltd. (Shanghai, China).

## 5. Conclusions

In the present study, DY80, an accession of Dongxiang wild rice, showed strong resistance to extreme heat stress. It will be very useful to utilize the elite genes or alleles, including upregulated genes in the pathway of unfolded protein binding, downregulated genes in the pathways of chlorophyll biosynthetic process, cysteine and methionine metabolism, photosystem I, photosystem II, and unchanged genes in the pathways of anchored components of the plasma membrane, cell wall biogenesis, and photosynthesis-antenna proteins, from Dongxiang wild rice for heat resistance genetic improvement in rice. Moreover, malic acid, stearic acid, and L-threonine might be causal metabolites for strong resistance to heat in Dongxiang wild rice. These results provide new insights into the mechanisms of heat resistance in rice. In conclusion, while this study advances our understanding of heat resistance in rice, addressing these limitations through functional validation, expanded genetic diversity, field trials, and multi-omics integration will be essential for developing heat-tolerant rice varieties to mitigate the impacts of climate change on rice production.

## Figures and Tables

**Table 1:** Summary of differential metabolites identified in DY80 and R974.

Group	Total	Upregulated	Downregulated
DY80 vs. DY80-CK	355	262	93
R974 vs. R974-CK	82	63	19

## Data Availability

The transcriptomic raw data have been made publicly available through the SRA database of NCBI with the submission ID PRJNA1240239. The metabolomic raw data are shown in Table S9. The data used during the current study are available from the corresponding author upon reasonable request.

## Supplementary Materials

The following supporting information can be downloaded at: https://www.mdpi.com/article/10.3390/plants14081192/s1, Figure S1: Correlation coefficients and PCA analysis among three biological replications of DY80 and R974 in transcriptomic data: (a) The correlation coefficients; (b) PCA analysis. PC1 represents the first principal component, and PC2 represents the second principal component. DXWR represents DY80, CK represents without heat treatment; Figure S2: Enrichment analyses of DEGs in various databases. (a) GO plot of DY80; (b) GO plot of R974; (c) COG plot of DY80; (d) COG plot of R974; (e) eggNOG plot of DY80; (f) eggNOG plot of R974; (g) KOG plot of DY80; (h) KOG plot of R974; Figure S3: The identical pathways in which DEGs enriched both in DY80 and R974 detected by GSEA analysis: (a), (c), and (e) are the trends of nucleosome, apoplast, and protein processing in the endoplasmic reticulum in DY80, while (b), (d), and (f) are the trends in R974. The horizontal axis represents the position information of the sorted gene set, with the black vertical lines indicating the genes within that GO Term/KEGG pathway. The vertical axis of the upper graph represents the dynamic enrichment score, with the green curve showing the enrichment score of the gene set at each position. The peak of the green curve corresponds to the enrichment score (ES) of the GO Term/KEGG pathway. If the ES value is positive, the genes enriched before the peak gene are considered core genes of the gene set, and the current function is indicated as an upward trend. If the ES value is negative, the genes enriched after the peak gene are considered core genes of the gene set, and the current function is indicated as a downward trend. The vertical axis of the lower graph represents the sorted quantity (log2FC), with the height of the black vertical lines indicating the size of the log2FC for the genes in the GO Term/KEGG pathway, corresponding to the red and blue annotations in the middle; Figure S4: The differential pathways in which DEGs enriched in DY80 and R974 detected by GSEA analysis: (a), (b), (c), (d), and (e) are the trends of five pathways in DY80, while (f), (g), and (h) are the trends of three pathways in R974; Figure S5: Correlation coefficients and PCA analysis among three biological replications of DY80 and R974 in metabolomic data. (A) The correlation coefficients; (B) PCA analysis. PC1 represents the first principal component, and PC2 represents the second principal component. DXWR represents DY80, and CK represents without heat treatment. Table S1: Transcriptome sequencing data after statistical analysis; Table S2: Statistics on data mapping; Table S3: DEGs identified in DY80; Table S4: DEGs identified in R974; Table S5: Summary of annotated DEGs identified in DY80 and R974; Table S6: Annotated differential metabolites in KEGG identified in DY80; Table S7: Annotated differential metabolites in KEGG identified in R974; Table S8: The primer sequences for qRT-PCR; Table S9: The metabolites detected in this study.

## Abbreviations

The following abbreviations are used in this manuscript:

COG	Clusters of orthologous groups of proteins
DEGs	Differential expressed genes
EC	Enzyme classification
FC	Fold change
FDR	False discovery rate
GO	Gene ontology
GSEA	Gene set enrichment analysis
IPCC	Intergovernmental panel on climate change
KEGG	Kyoto encyclopedia of genes and genomes
KO	KEGG ortholog database
Nr	NCBI non-redundant protein sequences
Nt	NCBI non-redundant nucleotide sequences
PCA	Principal component analysis
VIP	Variable importance in projection
Pfam	Protein family
qRT-PCR	Real-time quantitative PCR
ROS	Reactive oxygen species
TCA	Tricarboxylic acid tricarboxylic acid
UPLC-MS/MS	Ultra-performance liquid chromatography–tandem mass spectrometry
