# Supplementary figures and images for: Combined Analysis of Transcriptome and Metabolome Reveals the Heat Stress Resistance of Dongxiang Wild Rice at Seedling Stage
Source: Plants (Basel). 2025 Apr 11;14(8):1192. doi: 10.3390/plants14081192 (PMC12030080; doi:10.3390/plants14081192)

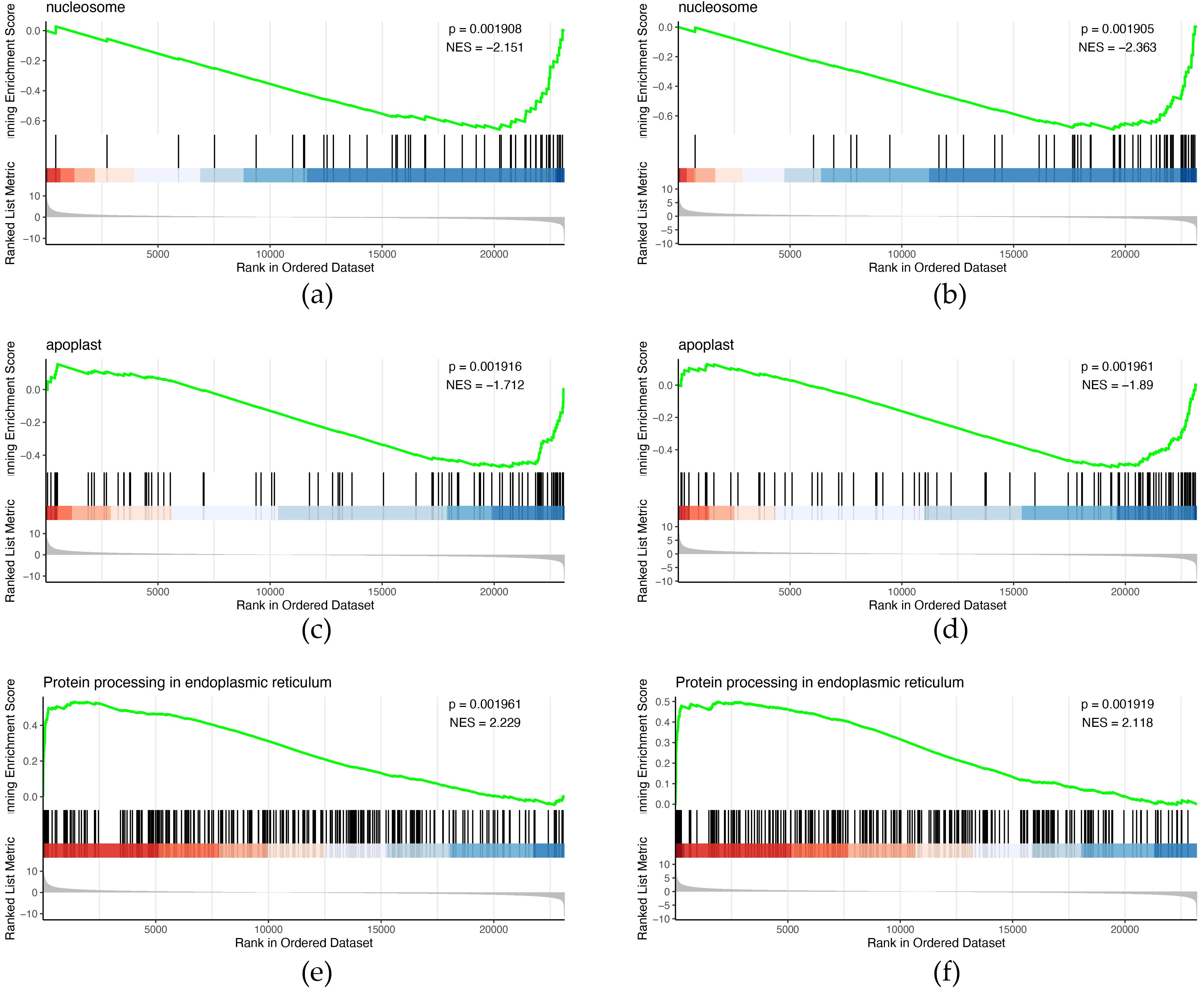

Supplement: Supplementary file 1 [file plants-14-01192-s001.zip › Supplementary figures and tables/Figure S3.jpg]

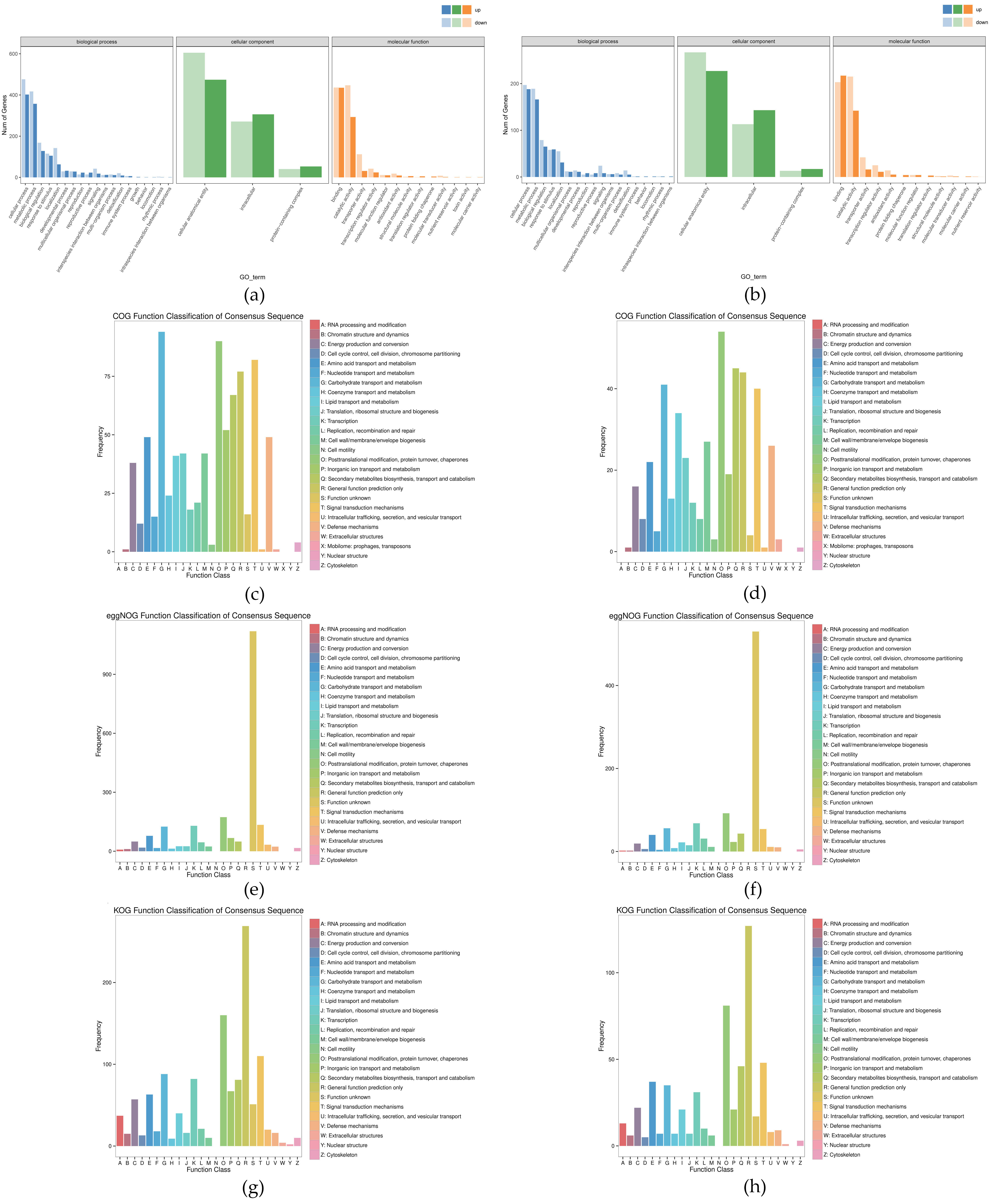

Supplement: Supplementary file 1 [file plants-14-01192-s001.zip › Supplementary figures and tables/Figure S2.jpg]

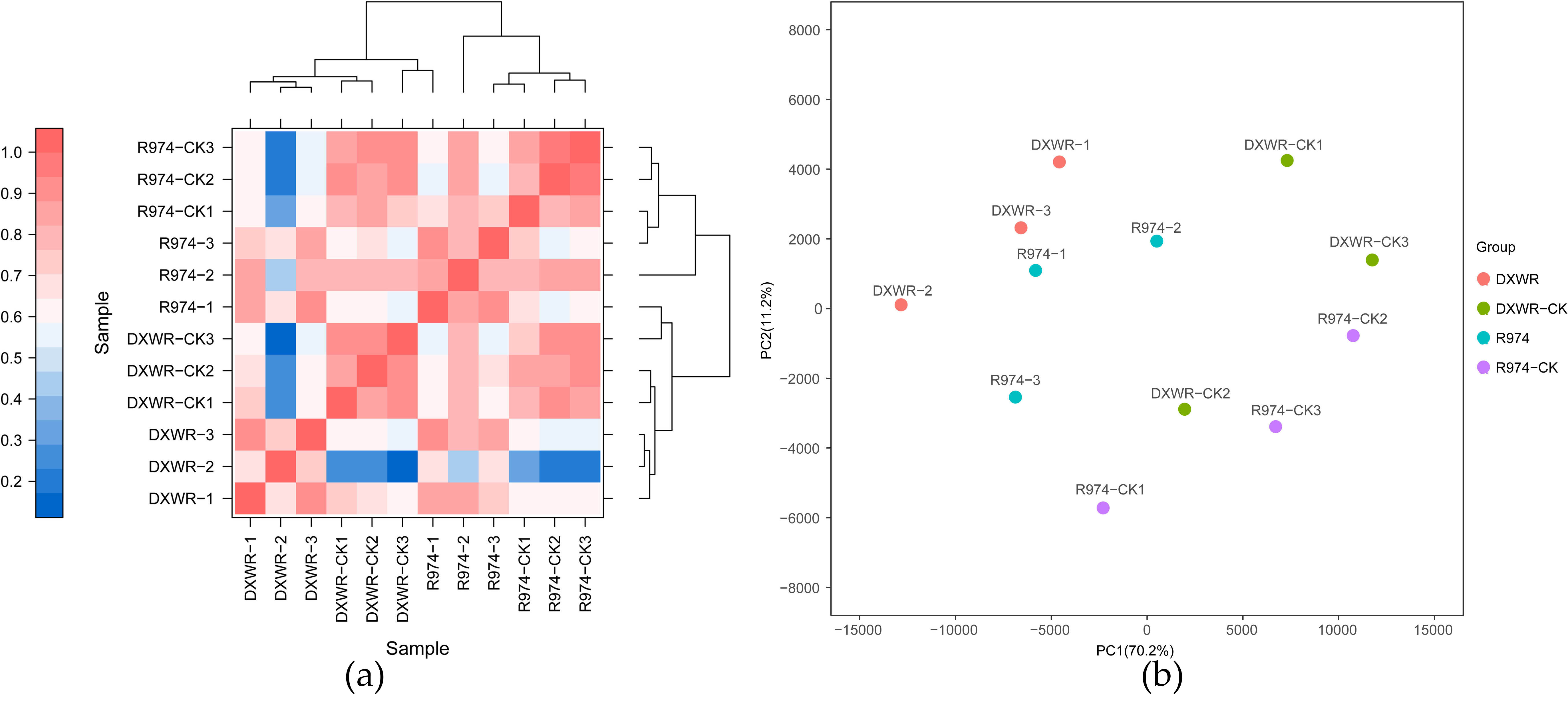

Supplement: Supplementary file 1 [file plants-14-01192-s001.zip › Supplementary figures and tables/Figure S1.jpg]

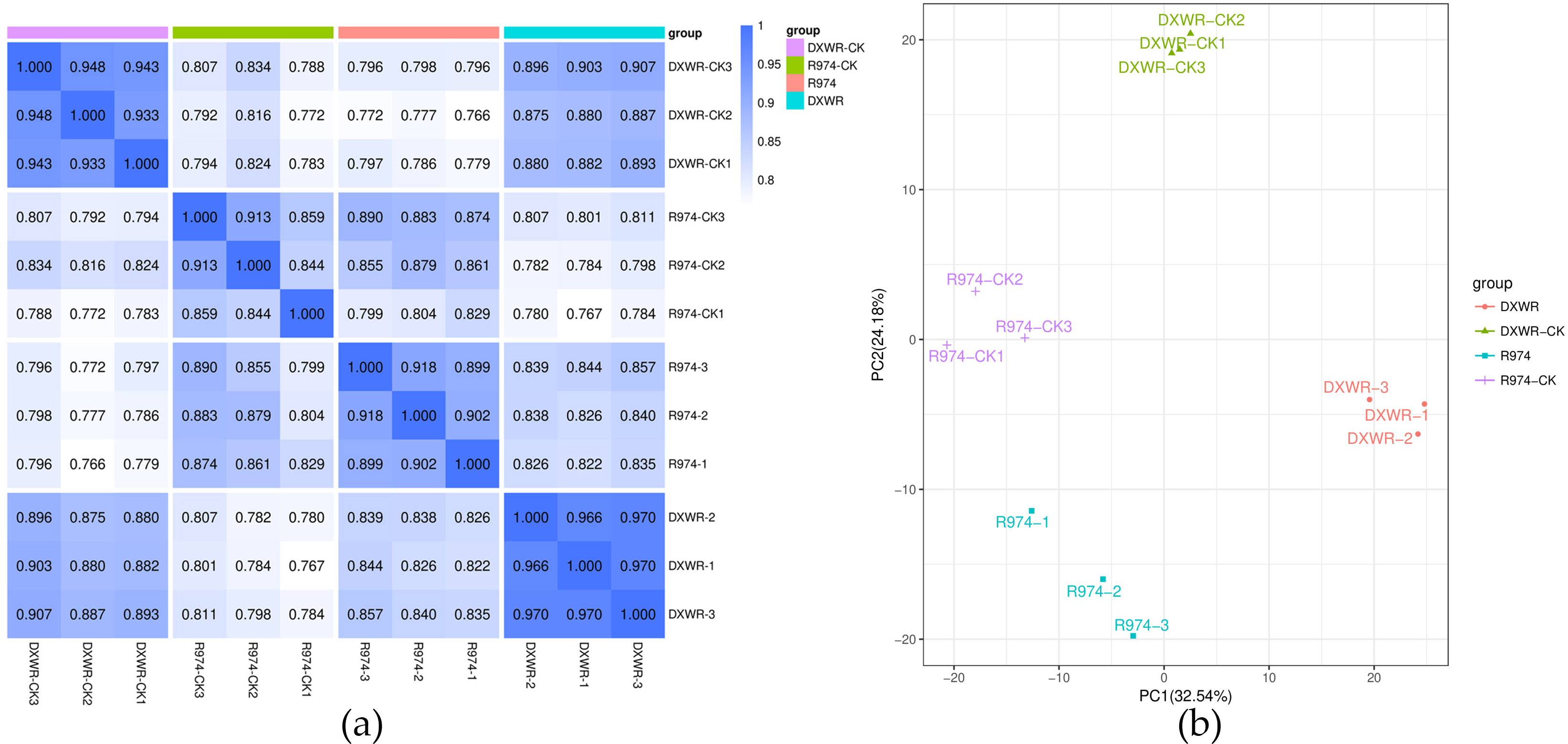

Supplement: Supplementary file 1 [file plants-14-01192-s001.zip › Supplementary figures and tables/Figure S5.jpg]

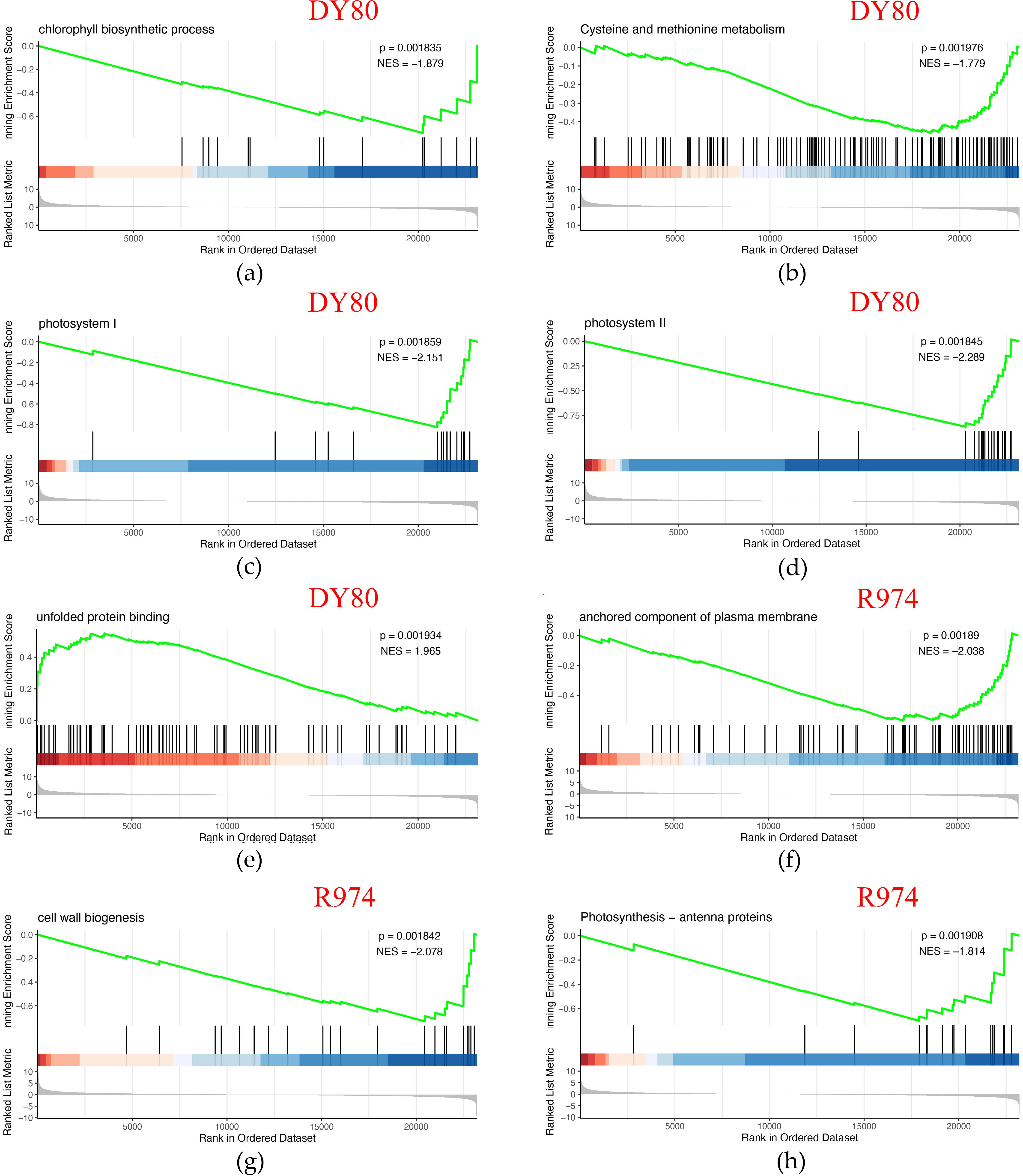

Supplement: Supplementary file 1 [file plants-14-01192-s001.zip › Supplementary figures and tables/Figure S4.jpg]
